# Association of *IL-16* gene polymorphisms with the risk of developing type 2 diabetes mellitus in the Chinese Han population

**DOI:** 10.1042/BSR20190821

**Published:** 2019-08-15

**Authors:** Fangxiao Cheng, Lu Liu, Hongli Zhang, Yi Zhu, Xiaohua Li, Hong Li

**Affiliations:** 1 Department of Endocrinology, Peking University First Hospital, Beijing 100034, China; 2 Department of Endocrinology, Seventh People’s Hospital of Shanghai University of TCM, Shanghai 200137, China; 3 Department of Endocrinology and Metabolism, Shanghai Tenth People’s Hospital, Tongji University School of Medicine, Shanghai 200072, China

**Keywords:** IL-16, PCR-RFLP, polymorphism, T2DM

## Abstract

**Objective:** The aim of the present study was to explore the genetic association of single nucleotide polymorphisms (SNPs) in interleukin-16 (*IL-16*) gene with type 2 diabetes mellitus (T2DM) susceptibility in a Chinese Han population.

**Methods:** In total, 133 T2DM patients and 127 healthy controls matched by age and gender were recruited in the case–control study. *IL-16* gene rs4778889 and rs11556218 polymorphisms were genotyped in the two groups via polymerase chain reaction-restriction fragment length polymorphism (PCR-RFLP) method. Differences in genotype and allele distributions between groups were compared by the χ^2^ test. All the comparisons were adjusted for age, gender, and body mass index (BMI) by logistic regression. The odds ratios (ORs) and 95% confidence intervals (CIs) were used to evaluate the association strength between *IL-16* gene polymorphism and T2DM risk.

**Results:** The TG genotype and G allele frequencies of rs11556218 increased remarkably in the case group than that in controls (45.86 vs 33.86%; 29.70 vs 20.87%), and the differences reached a significant level (*P*<0.05). After adjusting for age, gender, and BMI, the differences still reached a significant level (*P*<0.05). Rs11556218 TG genotype carriers had a 1.769-fold increased risk of developing T2DM (OR = 1.769, 95% CI = 1.045–2.994), and G allele was also associated with an increased risk of T2DM (OR = 1.639, 95% CI = 1.087–2.471). *IL-16* rs4778889 polymorphism showed no significant association with T2DM risk.

**Conclusion:** *IL-16* gene rs11556218 polymorphism was significantly associated with T2DM susceptibility in the Chinese Han population, while rs4778889 was not.

## Introduction

Type 2 diabetes mellitus (T2DM) is a complex metabolic disorder, characterized by elevated blood glucose and attenuated insulin action [1]. T2DM has been regarded as an important global public health problem [2]. In China, the onset age of T2DM shows a younger trend, and the morbidity has been ranked first in the world [3,4]. Substantial evidence demonstrate that T2DM can be attributed by lifestyle, environmental and genetic factors [5–8]. Although the etiology of T2DM is complex and needs further study, genetic factors have been identified to be an important cause, and an increasing number of candidate genes have been identified recent years [9,10]. Thus, investigating the candidate genes involved in the onset and development of T2DM may shed light on the molecular mechanisms underlying the disease.

Interleukin-16 (IL-16) is a pleotropic inflammatory cytokine which can be produced by activated T cells, B cells, monocytes, as well as cancerous cells. The human *IL-16* gene is located on chromosome 15q26.3. Up to now, numbers of single nucleotide polymorphisms (SNPs) have been identified in human *IL-16* gene, such as rs4778889, rs11556218, and rs4072111. The polymorphisms in *IL-16* gene may influence the expression and secretion of IL-16, which further result in relevant biological responses. The previous evidence has shown that *IL-16* gene polymorphisms play a crucial role in the pathogenesis of various diseases, such as osteoporosis, renal cell cancer, Alzheimer’s disease [11–13].

Although the molecular mechanism of T2DM is now unclear, the role of the inflammatory response in the pathogenesis of T2DM has been recently investigated [14,15]. As a proinflammatory factor, IL-16 is involved in the occurrence and development of inflammatory response, which participates in the progression of T2DM [16,17]. Additionally, a significant increase in serum IL-16 has been reported in patients with T2DM, suggesting its potential role in the development of T2DM [18]. Rs4778889 and rs11556218 are the two most-studied SNPs in previous researches, which showed a close association with the onset of several human diseases, such as cancers, osteoarthritis, coronary artery disease [11,19,20]. Furthermore, a remarkably decreased frequency of *IL-16* rs4778889 CC genotype has been detected in women with gestational diabetes mellitus (GDM), suggesting the genetic role of *IL-16* polymorphisms in GDM [21]. But the association of *IL-16* gene polymorphisms and T2DM susceptibility has not been investigated.

Therefore, a case–control study was conducted to clarify the relationship between *IL-16* gene rs4778889 and rs11556218 polymorphisms and T2DM susceptibility in a Chinese Han population. This work will provide a theoretical basis for early diagnosis and prevention of the disease.

## Materials and methods

### Study subjects

A total of 133 patients with T2DM were randomly recruited, who attended Seventh People’s Hospital of Shanghai University of TCM from April 2014 to July 2015. In total, 127 healthy individuals were recruited as control group, who came to the same hospital for a routine health check-up during the same time. The age and gender of participants in the control group were frequency-matched with the case group. All subjects were collected based on a rigorous set of criteria. The inclusion criteria for the cases were based on the American Diabetic Association (ADA) guidelines: individuals with a fasting blood glucose level ≥ 7 mmol/l, a 2-h postprandial blood glucose level ≥ 11.1 mmol/l or a glycated hemoglobin level ≥ 6.5%, and without a family history of diabetes [22]. Patients who suffered from other morbidities such as malnutrition, pancreatitis, anemia and malignant cancer were excluded from the study. For healthy controls, the inclusion criteria were as follows: (i) fasting blood glucose level < 7 mmol/l and a glycated hemoglobin level < 6.0%; (ii) no history of diabetes or other autoimmune disease in the degree relatives, and (iii) no hypertension. None of the subjects were receiving any antidiabetic, antihypertensive, or hypolipidemic drugs. A full clinical examination was performed. The demographic data including age, gender and Body Mass Index (BMI) and biochemical parameters as fasting blood glucose, total cholesterol, triglyceride, high-density lipoprotein (HDL) and low-density lipoprotein (LDL) were recorded.

The whole study idea was approved and supported by the Ethics Committee of Seventh People’s Hospital of Shanghai University of TCM. The sample collection was in accordance with the ethic criteria of National Human Genome Research. All participants were Chinese Han population, who had no blood relationship with each other. All subjects involved in the present paper were required to sign informed consents.

### Sample collection and genotyping

A total of 5 ml peripheral venous blood was collected from each individual, anticoagulated by 5% ethylene diamine tetra acetic acid (EDTA). Genomic DNA was obtained from peripheral leukocytes by the Takara Genome DNA Extraction Kit (Beijing Boiteke Corporation, Beijing, China), and stored at −20°C for standby application.

The target fragment for *IL-16* gene rs4778889 and rs11556218 polymorphisms was partially amplified using polymerase chain reaction (PCR) and genotyped by restriction fragment length polymorphism (RFLP) method. The primer sequences for rs4778889 and rs11556218 were designed by Primer Premier 5.0, and synthesized by Shanghai Sangon Biotech Co., Ltd (Table 1). The PCR procedures consisted of an initial degeneration at 95°C for 2 min, followed by 35 cycles of 95°C degeneration for 1 min, annealing at 60°C for 30 s, and extension at 72°C for 30 s, and finally extension at 72°C for 10 min. Then the PCR products of *IL-16* gene rs4778889 and rs11556218 polymorphisms were digested by the restriction enzymes *Ahd I* and *Nde I*, respectively, and the digested fragments were separated by electrophoresis on a 2% agarose gel and visualized by UV light. For rs4778889 polymorphism, samples producing a 280-bp band were typed as TT genotype, samples producing two bands of 246 and 34 bp were typed as CC genotype, and samples producing three bands of 280, 246, and 34 bp were typed as TC genotype. For rs11556218 polymorphism, samples producing a 171-bp band were typed as GG genotype, samples producing two bands of 147 and 24 bp were typed as TT genotype, and samples producing three bands of 171, 147, and 24 bp were typed as TG genotype.

**Table 1:** Primer sequences of *IL-16* gene rs4778889 and rs11556218 polymorphisms

Variations		Primer sequences
rs4778889	For.	5′-CTCCACACTCAAAGCCTTTTGTTCCTATGA-3′
	Rev.	5′-CCATGTCAAAACGGTAGCCTCAAGC-3′
rs11556218	For.	5′-GCTCAGGTTCACAGAGTGTTTCCATA-3′
	Rev.	5′-TGTGACAATCACAGCTTGCCTG-3′

### Statistical analysis

All statistical analyses of the present paper were performed using the PASW statistics 18.0 statistical software. The genotype and allele frequencies for *IL-16* gene rs4778889 and rs11556218 polymorphisms were calculated by direct counting. The Hardy–Weinberg equilibrium (HWE) of our study population was estimated via the chi-square test to assess the quality of the study sample. Chi-square test was employed to compare the genotype and allele distributions of rs4778889 and rs11556218 polymorphisms between groups, and all comparisons were adjusted for age, gender, and BMI by logistic regression. Odds ratios (ORs) and 95% confidence intervals (95% CIs) were used for the assessment of the association between *IL-16* gene polymorphisms and T2DM susceptibility. All *P*-values were two-tailed, and *P*-values less than 0.05 were considered as significant.

## Results

### Basic features

In the present study, 133 T2DM patients and 127 healthy controls were enrolled, and the age and gender of participants in the control group were frequency-matched with the case group (*P*=0.412, *P*=0.829). The demographic and medical data in the case and control groups are shown in Table 2. The values of BMI, fasting blood glucose, total cholesterol, and LDL were higher in the case group than that in controls, while the HDL was lower in cases, all differences reached significant level (*P*<0.05). No significant difference was detected for the value of triglyceride between case and control groups (*P*>0.05).

**Table 2:** Patients’ demographics and risk factors in T2DM patients

Variable	Cases (*n*=133)	Controls (*n*=127)	*P*
Age (years)	54.08 ± 7.23	53.33 ± 7.52	0.412
Female/male	52/81	48/79	0.829
BMI (kg/m^2^)	24.50 ± 4.20	23.13 ± 3.04	0.003
Fasting blood glucose (mmol/l)	8.90 ± 0.99	5.30 ± 0.58	<0.001
Total cholesterol (mmol/l)	5.38 ± 0.66	4.50 ± 0.55	<0.001
Triglyceride (mmol/l)	1.71 ± 0.61	1.57 ± 0.54	0.058
HDL (mmol/l)	1.30 ± 0.42	1.44 ± 0.34	0.002
LDL (mmol/l)	2.80 ± 0.33	2.69 ± 0.48	0.043

### Genetic association of *IL-16* gene polymorphisms with T2DM

Table 3 presented the genotype and allele distributions of *IL-16* gene rs4778889 and rs11556218 polymorphisms in both case and control groups. Via chi-square test, we noted that the genotype distributions of each SNP did not deviate from HWE in both case and control groups (*P*>0.05), demonstrating a good representativeness of our study population.

**Table 3:** Association of *IL-16* gene rs4778889 and rs11556218 polymorphisms with T2DM susceptibility

Genotype/Allele	Case, *n*=133 (%)	Control, *n*=127 (%)	*P*-value	OR (95% CI)	*P*^1^	OR (95% CI)^1^
rs4778889						
TT	79 (59.40)	73 (57.48)	-	-	-	-
TC	51 (38.35)	48 (37.80)	0.943	0.982 (0.592–1.629)	0.940	0.980 (0.582–1.649)
CC	3 (2.25)	6 (4.72)	0.277	0.462 (0.111–1.915)	0.274	0.444 (0.103–1.905)
T	209 (78.57)	194 (76.38)	-	-	-	-
C	57 (21.43)	60 (23.62)	0.549	0.882 (0.584–1.331)	0.538	0.876 (0.575–1.334)
*P_HWE_*	0.110	0.593				
rs11556218						
TT	63 (47.37)	79 (62.20)	-	-	-	-
TG	61 (45.86)	43 (33.86)	0.027	1.779 (1.066–2.968)	0.034	1.769 (1.045–2.994)
GG	9 (6.77)	5 (3.94)	0.154	2.257 (0.720–7.073)	0.114	2.584 (0.796–8.389)
T	187 (70.30)	201 (79.13)	-	-	-	-
G	79 (29.70)	53 (20.87)	0.021	1.602 (1.073–2.392)	0.018	1.639 (1.087–2.471)
*P_HWE_*	0.257	0.776				

^1^, Adjusted for age, gender and BMI.

As shown in Table 3, no significant difference was detected for *IL-16* gene rs4778889 polymorphism between case and control groups. For rs11556218 polymorphism, the TG genotype frequency increased significantly in the case group than that in controls (45.86 vs 33.86%, *P*<0.05), and individuals carrying TG genotype showed higher risk of T2DM (OR = 1.779, 95% CI = 1.066–2.968). Besides, the G allele of rs11556218 also showed a remarkable increasing trend in cases (29.70 vs 20.87%), and the differences reached a significant level (*P*<0.05). The G allele of rs11556218 polymorphism was associated with an increased risk of T2DM (OR = 1.602, 95% CI = 1.073–2.392).

In order to get an accurate result, we further took age, gender, and BMI as common confounders in the analysis of the association between *IL-16* gene polymorphisms and T2DM susceptibility. Adjusted results indicated that *IL-16* gene rs11556218 still showed significant association with T2DM susceptibility, and individuals carrying TG genotype and G allele showed higher risk to suffer from T2DM (OR = 1.769, 95% CI = 1.045–2.994; OR = 1.639, 95% CI = 1.087–2.471) (Table 3).

## Discussion

T2DM is regarded as a polygenic endocrine disease accompanied by glycometabolism disorders. T2DM has become a serious public problem especially in developing countries [23]. In recent years, with the prevalence of T2DM increasing year by year, cardiovascular morbidity and mortality have been on the rise, including hypertension, dyslipidemia, macrovascular and microvascular complications [24]. Up to now, a number of genetic factors have been reported to be associated with the onset of T2DM. Importantly, the crucial role of family history in the onset of T2DM has been widely reported which might be powerful than genetic factors, with the most likely explanation of that a family history reveals elements of lifestyle [25]. Therefore, in the present study, the enrolled T2DM patients had no family history.

IL-16 is a multifunctional proinflammatory cytokine, and exerts significant influence on the homeostasis of the immune and neuroendocrine systems, as well as the balance of proinflammatory/anti-inflammatory pathways. IL-16 has been suggested to play crucial role in several inflammatory diseases by promoting the secretion of cytokines, such as allergic rhinitis, inflammatory bowel disease (IBD) [26,27]. As is well-known, the role of the inflammatory response in the pathogenesis of T2DM has been confirmed. Recently, the role of *IL-16* gene polymorphisms in GDM has been reported [28], but the association of *IL-16* gene polymorphisms and T2DM susceptibility has not been investigated.

In the present study, we selected two common SNPs of IL-16 gene, namely rs4778889, associated with altered levels of gene expression, as well as rs11556218, representing an asparagine to lysine substitution in exon 6 of *IL-16* gene [19,29], to explore their potential relevance with the risk of T2DM in a Chinese Han population. Significant differences were detected for rs11556218 polymorphism between T2DM patients group and controls. The TG genotype and G allele frequencies increased significantly in the case group, suggesting rs11556218 TG genotype and G allele might be risk factors for the onset of T2DM. The comparisons were further adjusted for age, gender, and BMI by logistic regression. The results suggested that, compared with the TT genotype, the TG genotype carriers had 1.769-times risk of developing T2DM, and the G allele was still a risk factor for the onset of T2DM. The rs11556218 polymorphism is located on exon 6 of *IL-16* gene, resulting in an asparagine to lysine substitution [19]. Previous studies have confirmed that rs11556218 polymorphism shows a significant association with a number of human cancers [30,31]. Inflammation is known to be associated with increased risk of cancer, and patients with T2DM have an increased inflammatory state and are also at increased risk of cancer [32,33]. Besides, the significant association has been detected between rs11556218 polymorphism and several diseases associated with inflammation, such as systemic lupus erythematosus and knee osteoarthritis [19,34]. In view of the crucial role of the inflammatory response in the pathogenesis of T2DM, the association of rs11556218 polymorphism and T2DM was investigated in the present study. As expected, we found rs11556218 TG genotype carriers had a 1.769-fold increased risk of developing T2DM. These data suggested that *IL-16* rs11556218 polymorphism plays an important role in inflammation and that it includes T2DM, which in turn confirms the critical role of inflammation in T2DM. We failed to find any association between *IL-16* rs4778889 polymorphism and T2DM risk. Previous evidence has suggested that the frequency of *IL-16* rs4778889 CC genotype was decreased in women with GDM, it might because the difference of the study population in which only pregnant women were enrolled in the previous study and pregnancy may affect the immune system [21].

Some studies suggest that genetic mutations in the *IL-16* gene produce altered protein products with varying cytokine activity [35]. It is also reported that the increased expression of IL-16 in islets is related to the development of invasive insulitis, and neutralization of IL-16 might be a potential therapy for the prevention of diabetes [36]. Thus, we speculated that the contribution of *IL-16* gene polymorphism to T2DM may arise from altered levels of IL-16 production or activity. Further studies are needed to verify the hypothesis.

There are some limitations in the present study. All participants enrolled in the present study were from a single hospital and the study sample was relatively small, it cannot role out the possibility of selection bias. But both case and control groups did not deviate from HWE for each SNP, indicating the possibility is minimal. Besides, T2DM is a complex disease which can be affected by both genetic and environmental factors. In the present study, gene–environment interaction cannot be assessed as a result of a lack of available data. Further gene–environment interaction analysis may provide strong evidence of *IL-16* polymorphisms in the etiology of T2DM. Maybe a follow-up epigenetic study can be conducted. Additionally, the expression level of IL-16 in T2DM patients was not measured in the present study. The effects of these polymorphisms on IL-16 expression can be clarified as well as the mechanisms by which these polymorphisms contribute to T2DM in further researches.

In conclusion, the present study suggested a significant association between *IL-16* gene rs11556218 polymorphism and T2DM susceptibility in the Chinese Han population, and TG genotype carriers showed higher risk to suffer from T2DM. Further well-designed studies involving various populations are warranted to verify the present results.

## Abbreviations

ADA: American Diabetic Association
BMI: body mass index
EDTA: ethylene diamine tetra acetic acid
GDM: gestational diabetes mellitus
HDL: high-density lipoprotein
HWE: Hardy–Weinberg equilibrium
IBD: inflammatory bowel disease
IL-16: interleukin-16
LDL: low-density lipoprotein
OR: odds ratio
PCR: polymerase chain reaction
RFLP: restriction fragment length polymorphism
SNP: single nucleotide polymorphism
T2DM: type 2 diabetes mellitus
95% CI: 95% confidence interval
